# Value Co‐Creation Between Nurses and Generative Artificial Intelligence: A Grounded Theory Study

**DOI:** 10.1155/jonm/8980490

**Published:** 2026-08-28

**Authors:** Yingying Jia, Wei Zhu, Yan Cai, Yan Jiang

**Affiliations:** ^1^ Center of Gerontology and Geriatrics, West China Hospital, Sichuan University/West China School of Nursing, Sichuan University, Chengdu, Sichuan, China, scu.edu.cn; ^2^ Evidence-Based Nursing Center, West China Hospital, Sichuan University, Chengdu, Sichuan, China, scu.edu.cn; ^3^ Sichuan Provincial Engineering Research Center of Medical Nursing Equipment and Materials, Chengdu, Sichuan, China, scu.edu.cn; ^4^ Department of Neurosurgery, West China Hospital, Sichuan University, Chengdu, Sichuan, China, scu.edu.cn; ^5^ Department of Nursing, West China Hospital, Sichuan University/West China School of Nursing, Sichuan University, Chengdu, Sichuan, China, scu.edu.cn

**Keywords:** generative artificial intelligence, grounded theory, human–GenAI collaboration, nursing informatics, qualitative research, value co-creation

## Abstract

**Background:**

The role of generative artificial intelligence (GenAI) in nursing is increasingly shifting from a support tool toward a collaborative partner. However, the mechanisms underlying nurse–GenAI value co‐creation (N‐GAIVC) remain undertheorized.

**Aim:**

To develop a theoretical framework for N‐GAIVC.

**Methods:**

This study used a Straussian grounded theory approach. Between January and April 2026, 27 clinical nurses and nurse managers from hospitals across China were recruited using purposive, snowball, and theoretical sampling. Semistructured in‐depth interviews and online textual data from Chinese social media platforms were analyzed. Data analysis followed the constant comparative method, with iterative open, axial, and selective coding conducted to generate and refine categories. Theoretical saturation was assessed through supplementary interviews and considered achieved when no new categories or relationships emerged.

**Results:**

A theoretical framework of nurse–GenAI value co‐creation was developed, comprising three integrative domains. Situated engagement conditions included engagement drivers, appraising GenAI fit, and safe‐use scaffolding. Professional orchestration in practice involved selective delegation, prompting, verification, contextualization, feedback, peer diffusion, and implementation embedding. Conditional value outcomes encompassed both co‐creation and destruction and recursively shaped subsequent engagement, safeguards, and nurse–GenAI co‐evolution.

**Conclusions:**

Nurse–GenAI value co‐creation is a situated, professionally orchestrated, and adaptive process. Value arises not from GenAI use alone, but from nurses’ appraisal of task fit and risk, active orchestration of GenAI contributions, and alignment with nursing expertise, human‐centered care, and organizational safeguards.

**Implications for Nursing Management:**

The framework provides a practical roadmap for responsible GenAI integration in nursing practice. Nurse managers should assess task suitability and risk; strengthen nurses’ GenAI literacy and critical evaluation; establish training, governance, accountability, and validation mechanisms; and support nurse‐led feedback and workflow embedding. These measures can enhance value while mitigating overreliance, added workload, and erosion of relational and professional capabilities.

## 1. Introduction

Generative artificial intelligence (GenAI) is increasingly being integrated into nursing practice, offering new opportunities to address emerging healthcare challenges [1, 2]. Effective GenAI integration depends not only on technological adoption but also on nurses’ active interpretation, verification, and contextualization of GenAI‐generated outputs, highlighting the importance of collaborative rather than purely technological integration [3–6].

Service‐dominant logic offers a theoretical lens for understanding nurse–technology value co‐creation, proposing that value emerges through resource integration and interactions among actors within service ecosystems [7, 8]. From a service‐dominant logic perspective, GenAI can be conceptualized as an operant resource that contributes computational capabilities, information processing, and decision‐support capacity, while nurses contribute contextual understanding, professional judgment, and accountability through human–GenAI value co‐creation [7–9]. Through the integration of these complementary resources, nurse–GenAI collaboration has the potential to generate clinical and experiential value.

Emerging empirical evidence further demonstrates how nurse–GenAI collaboration can generate clinical and experiential value in healthcare settings. Evidence from a randomized controlled trial demonstrated that a nurse–GenAI collaborative model improved communication efficiency, patient satisfaction, and perceived empathy while reducing patients’ negative emotions compared with nurse‐only interactions [10]. Similarly, GenAI has the potential to reduce documentation burden by integrating multimodal patient data in real time, enabling nurses to focus on direct care while facilitating informed clinical decision‐making [11].

Nevertheless, the benefits of nurse–GenAI collaboration are not guaranteed. Human–GenAI collaboration represents a complex sociotechnical process in which outcomes are influenced by how GenAI capabilities are aligned with clinical tasks, workflows, and human decision‐making [12–14]. GenAI integration may initially disrupt workflows before generating productivity gains, reflecting a dynamic process of adaptation during technology‐enabled transformation [15]. Evidence suggests that human–GenAI teams do not consistently outperform either humans or GenAI alone [14]. For example, Wu et al. reported that GenAI‐assisted clinicians achieved similar diagnostic accuracy to standalone GenAI (58% vs. 60%) and higher accuracy than clinicians without GenAI assistance (58% vs. 27%) [12]. A recent meta‐analysis further found that human–GenAI combinations did not consistently outperform the best‐performing human or GenAI systems alone across different task contexts [16].

These findings indicate that human–GenAI integration does not automatically guarantee expected benefits and may not consistently generate positive outcomes across different contexts [14]. Potential mechanisms include cognitive biases such as anchoring bias, which may hinder clinicians’ critical evaluation of information during decision‐making [17, 18]. Nurses may either over‐rely on inaccurate GenAI outputs or fail to appropriately incorporate useful recommendations, undermining effective collaborative decision‐making.

Despite growing interest in GenAI applications in nursing, existing research remains largely exploratory, focusing on adoption, implementation challenges, and preliminary evaluations of clinical applications [19, 20]. Limited attention has been paid to the underlying mechanisms through which human, technological, and organizational factors interact to shape value co‐creation and value destruction in nurse–GenAI collaboration. Consequently, the conditions shaping whether GenAI integration produces beneficial or detrimental outcomes remain poorly understood, representing a critical knowledge gap in nursing informatics research [9, 20]. Furthermore, nurses and healthcare leaders may experience GenAI integration differently due to their distinct clinical and organizational roles [21, 22]. Understanding these perspectives is therefore essential for developing a comprehensive framework explaining how nurse–GenAI value co‐creation emerges and evolves.

To investigate this complex and multidimensional phenomenon, this study used a Straussian grounded theory approach guided by Corbin and Strauss. This study aims to develop a data‐driven theoretical framework explaining how clinical nurses and nursing managers co‐create value with GenAI and how value destruction may occur. The findings are expected to extend nursing informatics and GenAI application research, providing theoretical and practical guidance for the safe, standardized, and effective integration of GenAI in clinical nursing practice.

## 2. Methods

### 2.1. Design

Employing a Straussian grounded theory approach, this study aimed to develop an explanatory framework for nurse–GenAI value co‐creation processes. Unlike thematic description, which primarily identifies patterns within participants’ accounts [23], grounded theory seeks to explain how and why processes develop by examining conditions, actions/interactions, and consequences [24].

### 2.2. Sample and Recruitment

The study’s sampling strategy was guided by grounded theory principles, utilizing purposive, snowball, and theoretical sampling to achieve sample diversity and conceptual depth [24]. Sample diversity was sought across key contextual characteristics, including gender, age, education level, clinical department, years of nursing experience, professional title, hospital tier, and frequency of GenAI use.

Both clinical nurses and nursing managers were included. Eligibility criteria were as follows: (1) participants were required to have used GenAI for work‐related clinical, educational, research, or management tasks for at least three times per week; (2) clinical nurses were additionally required to be registered nurses with at least 1 year of clinical experience, whereas nurse managers were required to have at least 5 years of nursing management experience; and (3) willingness to participate and provide informed consent. Exclusion criteria were as follows: (1) nursing interns, visiting/training nurses; (2) individuals who were unable to complete the interview because of communication difficulties.

Recruitment proceeded through three iterative stages. First, purposive sampling was conducted based on predefined inclusion and exclusion criteria. Participants were recruited through on‐site recruitment at West China Hospital of Sichuan University and online advertisements on Chinese social media platforms, with eligibility verified based on professional role, clinical experience, GenAI exposure, and valid nursing credentials. Second, snowball sampling was employed, whereby enrolled participants recommended nurses with diverse clinical backgrounds and GenAI experiences. Third, as data collection and analysis proceeded concurrently, recruitment became increasingly theory‐driven. Early analysis generated detailed accounts of nurses’ delegation of cognitive tasks to GenAI and their verification and refinement of GenAI‐generated outputs, whereas the categories concerning organizational readiness and nurses’ participation in shaping GenAI implementation remained less developed. Theoretical sampling therefore focused particularly on nurse managers with experience in staff training, workflow coordination, resource allocation, and digital‐system implementation, as well as clinical nurses working in different organizational and technological contexts. Subsequent comparisons between accounts of value co‐creation and value destruction further guided recruitment across hospital contexts, levels of clinical responsibility, and frequencies of GenAI use to clarify how clinical risk, output verification, workload, accountability, and organizational support shaped nurse–GenAI interaction outcomes. Recruitment continued until theoretical saturation was achieved, defined as the point at which additional data no longer generated new conceptual properties or relationships among categories [24].

### 2.3. Data Collection

Data were collected through semistructured, in‐depth interviews and supporting digital textual data.

#### 2.3.1. Semistructured Interviews

Guided by grounded theory principles and semistructured interviewing methods, an interview guide was developed. The interview guide was refined based on expert feedback in nursing informatics. To ensure the clarity, relevance, and feasibility of the questions, three individual pilot interviews were conducted with two clinical nurses and one nursing manager. These pilot interviews were used to refine the wording, sequence, and probing questions in the interview guide; the pilot data were not included in the formal analysis. The specific interview guide can be found in Supporting Table S1 in Appendix A.

Between January and April 2026, the principal investigator (YYJ, PhD candidate) conducted semistructured, one‐on‐one interviews in private settings to ensure confidentiality. Prior to each formal interview, the researcher established initial rapport with participants through brief informal communication; explained the study purpose, procedures, confidentiality protections, and voluntary participation requirements; and obtained informed consent before participation. Interview appointments were then scheduled based on participants’ availability to minimize disruption to clinical work. Each participant completed one primary interview, with each session lasting 30–60 min. Brief follow‐up contacts were conducted when clarification of ambiguous accounts or emerging interpretations was required. Interview questions were open‐ended (e.g., “Could you describe a specific instance where you and GenAI successfully solved a clinical or administrative problem?”) and supplemented by exploratory probes (e.g., “Please elaborate,” “How did the hospital environment influence your GenAI usage?”). All interviews were audio‐recorded with informed consent and transcribed verbatim within 24 h. Member checking was conducted during the data collection process.

Data collection was supported by research memos and daily digital records. These archives consisted of the researcher’s daily reflexive journals, field observation notes, and verbatim transcriptions. Reflective memos and regular debriefing sessions with the supervisory team were used to refine interview questions and support concurrent data analysis. The interview guide was iteratively refined throughout the study, informed by reflective journaling and regular debriefing sessions with the supervisory team.

#### 2.3.2. Supplementary Online Data

To enhance data triangulation, supporting online textual data related to nurses’ GenAI use were collected from Chinese social media platforms. The dataset covered the period from January 2022 to April 2026. These online data were integrated with interview data to facilitate category development and assessment of theoretical saturation during grounded theory analysis. Detailed platform information and keyword strategies are provided in Table S2 in Appendix A.

### 2.4. Ethical Considerations

The study protocol was approved by the Ethics Committee on Biomedical Research, West China Hospital of Sichuan University (Approval number: 202609), and conducted in accordance with the Declaration of Helsinki. All participants were informed about the study purpose, procedures, and their right to withdraw at any time, and provided written or electronic informed consent prior to participation. Participant identities were protected through anonymized coding. Clinical nurses and nursing managers were assigned codes (N1, N2, etc., and M1, M2, etc., respectively). Audio recordings and transcripts were securely stored on encrypted, password‐protected devices accessible only to the research team. Supporting online textual data were collected only from publicly available social media posts and involved no direct interaction with users. Before analysis and reporting, all online data were anonymized. Usernames, personal identifiers, institutional names, and other potentially identifiable details were removed. Any quotations from online posts were presented in anonymized form (O1, O2, etc.).

### 2.5. Data Analysis

Data management and organization were facilitated by NVivo 12 software. Data collection and analysis were conducted concurrently through a constant comparative process comprising open, axial, and selective coding. Interview data and supporting online texts were integrated during coding and analyzed through constant comparison. During open coding, transcripts and online texts were examined line by line to identify meaningful incidents and generate initial concepts, which were subsequently grouped into broader categories. Axial coding focused on exploring relationships among categories and subcategories by examining conditions, contexts, interactions, and consequences. Selective coding was then conducted to identify the core category and integrate all categories into a coherent theoretical model. Theoretical sampling was applied iteratively throughout the analytic process to address conceptual gaps and refine emerging relationships. The coding process and category integration are presented in Supporting Tables S3–S5.

To enhance analytical rigor, initial coding was conducted by a three‐member research team comprising a senior qualitative researcher and two trained researchers. Coding interpretations and discrepancies were examined through iterative discussions until consensus was reached. An audit trail was maintained throughout the analysis and included original interview transcripts, online textual data, NVivo coding structures, analytic memos, and records of category development. Coding decisions and modifications were documented to ensure that the progression from initial concepts to subcategories, main categories, and the core category remained traceable to the original data. Credibility and confirmability were further supported through peer debriefing and reflexive discussions within the research team.

### 2.6. Rigor and Trustworthiness

The rigor and trustworthiness of this study were addressed through strategies supporting credibility, dependability, confirmability, and transferability, consistent with established qualitative research principles [25]. Credibility was supported through data‐source triangulation by comparing patterns, variations, and negative cases across interviews with clinical nurses and nurse managers and supporting online textual data, thereby helping to refine and challenge emerging interpretations. During data collection, ambiguous accounts and emerging interpretations were clarified through brief follow‐up contacts and participant checking. Synthesized findings were also discussed with 10 participants to assess whether the emerging interpretations accurately reflected their experiences. Dependability and confirmability were strengthened through team‐based coding, peer debriefing, reflexive discussions, and a documented audit trail of analytic decisions and category development. To support reflexivity, the principal investigator kept a reflexive journal documenting assumptions, methodological decisions, and reflections throughout data collection and analysis. Regular team discussions were used to examine how the researchers’ clinical and technological backgrounds might influence interpretation and to ensure that the categories remained grounded in the data. Transferability was supported through detailed descriptions of participant characteristics, clinical and organizational contexts, data sources, and analytic procedures, together with a diverse sample of clinical nurses and nurse managers. After the first 22 interviews had generated a preliminary theoretical framework, five additional interviews were conducted to assess theoretical saturation. Constant comparison indicated that these interviews generated no new categories, properties, dimensions, or relationships among categories, and the research team reached consensus that theoretical saturation had been achieved.

## 3. Results

### 3.1. Participant Sample and Data Sources

The 27 participants included 19 clinical nurses and 8 nurse managers, as detailed in Table 1. Of these, 23 (85.19%) were female and 13 (48.15%) had more than 10 years of work experience. Seventeen participants (62.96%) reported very frequent GenAI use (≥ 16 times/week). The mean age of the participants was 39.48 ± 9.33 years. The mean interview duration was 40 min.

**TABLE 1:** Participant characteristics (*n* = 27).

Variable		Frequency (n)	Percentage (%)
Gender	Male	4	14.81%
	Female	23	85.19%

Region of China	Eastern China	12	44.44%
	Central China	8	29.63%
	Western China	7	25.93%

Educational level	College degree or below	4	14.81%
	Bachelor’s degree	12	44.44%
	Master’s degree	8	29.63%
	Doctoral degree	3	11.11%

Professional title	Junior	11	40.74%
	Intermediate	10	37.04%
	Senior	6	22.22%

Work experience (years)	< 5	6	22.22%
	5–10	8	29.63%
	> 10	13	48.15%

Department	Intensive care unit	3	11.11%
	Emergency department	2	7.41%
	Cardiac rehabilitation	9	33.33%
	Other departments	13	48.15%

Frequency of GenAI use per week	Rarely used (3–5 times/week)	2	7.41%
	Occasionally used (5–9 times/week)	3	11.11%
	Frequently used (10–15 times/week)	5	18.52%
	Very frequently used (≥ 16 times/week)	17	62.96%

A total of four forum threads and 125 relevant interactive comments were included in the analysis. These online data complemented the interviews by capturing nurses’ spontaneous expressions of experiences about GenAI use in naturalistic online settings.

### 3.2. Overview of the Nurse–GenAI Value Co‐Creation Framework

**FIGURE 1:**
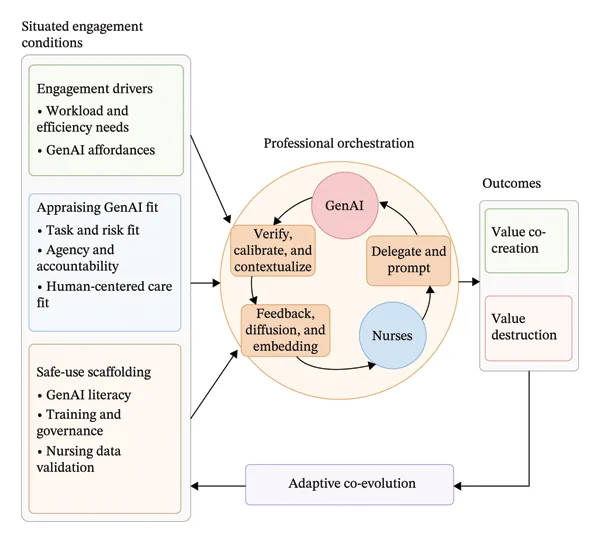
Theoretical framework for N‐GAIVC. Note. N‐GAIVC: nurse–GenAI value co‐creation. Selected category labels were shortened in the figure for visual clarity.

Line‐by‐line open coding generated 407 initial concepts, which were grouped into 39 open categories. Through axial coding, these categories were consolidated into 14 subcategories and 6 main categories. During selective coding, the six main categories were integrated around the core category of “nurse‐led GenAI orchestration for situated nursing value co‐creation” and further organized into three integrative domains: situated engagement conditions, professional orchestration in practice, and conditional outcomes and adaptive co‐evolution. As shown in Table 2 and Figure 1, situated engagement conditions comprised engagement drivers, appraising GenAI fit, and safe‐use scaffolding. These conditions shaped why nurses engaged with GenAI, how they determined whether it was appropriate for particular nursing tasks, and what safeguards were required for responsible use. Professional orchestration in practice represented the central action/interaction mechanism through which nurses delegated tasks, refined prompts, verified and contextualized outputs, and supported feedback and implementation. These interactions generated conditional value outcomes, which recursively reshaped subsequent engagement, task boundaries, governance arrangements, and nurse–GenAI co‐evolution. In Appendix A, Supporting Table S3 illustrates the progression from raw data to initial concepts and open categories; Supporting Table S4 details the axial integration of the 14 subcategories into 6 main categories; and Supporting Table S5 presents their selective integration within the three integrative domains and around the core category.

**TABLE 2:** Core category, integrative domains, main categories, and subcategories of nurse–GenAI value co‐creation.

Core category	Integrative domains	Main categories (MC)	Subcategories (SC)
Nurse‐led GenAI orchestration for situated nursing value co‐creation	I. Situated engagement conditions	MC1. Engagement drivers	SC1. Workload and efficiency needs SC2. Perceived GenAI affordances and role opportunities
		MC2. Appraising GenAI fit	SC3. Task codifiability and clinical risk SC4. Agency, accountability, and explainability SC5. Human‐centered care fit
		MC3. Safe‐use scaffolding	SC6. GenAI literacy and critical evaluation SC7. Training, infrastructure, and governance SC8. Nursing‐specific data and real‐world validation
	II. Professional orchestration in practice	MC4. Professional orchestration	SC9. Selective delegation and prompting SC10. Output verification and contextualization SC11. Feedback, diffusion, and implementation embedding
	III. Conditional outcomes and adaptive co‐evolution	MC5. Value outcomes	SC12. Value co‐creation SC13. Value destruction
		MC6. Adaptive co‐evolution	SC14. Adaptive feedback and nurse–GenAI co‐evolution

### 3.3. Situated Engagement Conditions

This domain describes the conditions under which nurses considered, appraised, and prepared for engagement with GenAI. It comprised three main categories: engagement drivers, appraising GenAI fit, and safe‐use scaffolding.

#### 3.3.1. Engagement Drivers

Engagement drivers captured the practice pressures and perceived technological possibilities that prompted nurses to consider or begin using GenAI. This main category comprised workload and efficiency needs and perceived GenAI affordances and role opportunities.

##### 3.3.1.1. Workload and Efficiency Needs

The subcategory focuses on practice pressures that made GenAI attractive to nurses. Interviewees reported that night work, documentation, and research‐related requirements added to already demanding workloads. These pressures created a practical impetus for nurses to seek tools that could ease workload intensity and improve work efficiency.

N5: “I really don’t want to work the night shift; I just feel like it would be great if a robot or GenAI system could take my place. Plus, a robot is—at the very least—just a hunk of metal, so it can take a beating.”

N14: “Hospitals now have extremely high research requirements for nurses… GenAI is the only ‘research mentor’ I can hire for free.”

##### 3.3.1.2. Perceived GenAI Affordances and Role Opportunities

The subcategory relates to what nurses believed GenAI could enable in practice and professional development. Participants described GenAI as a source of rapid nursing information, educational support, drafting assistance, and new opportunities for technology‐enabled practice. They generally positioned GenAI as augmenting nurses’ capabilities rather than replacing the profession, while recognizing that the ability to work with GenAI could influence future professional roles.

N6: “The GenAI guide for pediatric influenza nursing care is detailed and comprehensive.”

M5: “GenAI will mainly augment rather than replace nurses. Future nurses will be increasingly freed from efficiency‐oriented repetitive work and redirected toward inefficient but high‐value domains requiring breakthrough thinking.”

#### 3.3.2. Appraising GenAI Fit

Appraising GenAI fit captured how nurses determined whether, when, and to what extent GenAI was appropriate for a particular nursing activity. This appraisal encompassed task characteristics and clinical risk, professional agency and accountability, and compatibility with human‐centered care.

##### 3.3.2.1. Task Codifiability and Clinical Risk

The subcategory focuses on nurses’ assessment of whether tasks were sufficiently standardized, text‐based, and low risk for GenAI involvement. Participants distinguished information‐processing and preparatory work from embodied, unpredictable, or high‐stakes clinical activities. GenAI was therefore considered more appropriate for standardized, text‐based, and preparatory tasks than for hands‐on procedures or high‐risk clinical activities.

N9: “Nurses frequently use GenAI for tasks such as checking drug usage and side effects, interpreting lab results, and drafting handover notes; however, GenAI plays no role in actual hands‐on nursing procedures at present.”

N1: “Although GenAI can assist diagnosis, medical decision‐making must still integrate patients’ individual differences, emotional care needs, and complex clinical conditions. For example, tactile feedback and emergency response during surgical procedures rely on nurses’ experiential knowledge.”

##### 3.3.2.2. Agency, Accountability, and Explainability

The subcategory relates to the professional, ethical, and legal boundaries that preserve nurses’ authority and responsibility when working with GenAI. Participants emphasized that GenAI should not determine clinical actions independently or shift accountability away from human professionals. Concerns about black‐box reasoning, limited contextual awareness, and unclear liability reinforced the need to define nurses as final professional decision‐makers rather than passive executors of machine‐generated recommendations.

M1: “No matter how smart the GenAI is, nurses need strong clinical judgment to serve as a safeguard. It doesn’t understand ward interpersonal relationships or the local healthcare environment. Without solid professional knowledge, it’s easy to be misled by it.”

M8: “Technological bottlenecks and ethical dilemmas remain important, including immature tactile‐feedback technology, black‐box medical decision‐making, and legal gaps in liability for accidents.”

##### 3.3.2.3. Human‐Centered Care Fit

The subcategory focuses on whether GenAI use preserved the relational, emotional, and embodied foundations of nursing. Participants maintained that empathy, interpersonal trust, sensitivity to subtle changes, and physical responsiveness could not be reproduced through algorithmic outputs. GenAI was considered valuable when it created space for human connection, but problematic when it displaced or weakened person‐to‐person care.

N1: “At the core of nursing lies person‐to‐person care, which involves empathy, human warmth, adaptability, and physical dexterity—qualities that GenAI and robots find truly difficult to replicate.”

#### 3.3.3. Safe‐Use Scaffolding

Safe‐use scaffolding captured the individual and organizational resources that enabled or constrained responsible nurse–GenAI collaboration. It included nurses’ GenAI literacy, organizational training and governance, and access to nursing‐specific data and real‐world validation.

##### 3.3.3.1. GenAI Literacy and Critical Evaluation

The subcategory focuses on nurses’ capacity to use GenAI safely and critically. Interviewees reported that effective use required not only nursing knowledge but also digital competence, prompting skills, and the ability to judge whether GenAI‐generated information was trustworthy and clinically appropriate. Because GenAI tools were rapidly updated, participants also viewed GenAI literacy as an evolving capability that required continuous learning rather than one‐time technical training.

M3: “Nursing practitioners need a solid foundation in medical knowledge. They also need broad technical skills, including computer operation, data analysis, and GenAI principles and applications. Nurses must continuously improve their competencies and adapt to technological change.”

M8: “There are so many large language models, and they are constantly being updated. Even basic GenAI knowledge and skills change very quickly. Younger nurses learn these things more easily, but for older nurses, it is much more difficult to keep up.”

##### 3.3.3.2. Training, Infrastructure, and Governance

The subcategory relates to the institutional arrangements required to move GenAI from informal individual use into responsible nursing practice. Participants called for structured training, protected learning opportunities, technical infrastructure, clear accountability, and sustained financial and managerial support. Without these arrangements, implementation could remain fragmented and nurses could be expected to use rapidly changing systems without adequate preparation.

M5: “GenAI brings anxiety. We often feel that we are always facing something new that we do not understand, and there is a fear of being left behind or becoming less efficient than others. Hospitals should provide structured training programs to enhance nurses’ GenAI competencies.”

M6: “Our hospital has developed a nursing‐specific large language model and integrated it into the electronic medical record system. However, developing such a system requires substantial financial investment, and continuous resources are also needed for system optimization, maintenance, and staff training.”

##### 3.3.3.3. Nursing‐Specific Data and Real‐World Validation

The subcategory focuses on the contextual relevance and demonstrated performance of GenAI systems used in nursing. Participants noted that models developed primarily for medicine or general use did not necessarily reflect nursing knowledge, terminology, workflows, or patient interactions. They therefore emphasized nursing‐specific development and evaluation under real‐world conditions rather than reliance on technical performance alone.

N11: “Currently, the hospital has developed a dedicated GenAI model based on medical expertise and embedded it into the electronic medical record system to assist physicians with clinical documentation. However, a comparable model adapted to the nursing profession has not yet been developed.”

### 3.4. Professional Orchestration in Practice

Professional orchestration constituted the central action/interaction mechanism of the framework. Nurses translated general GenAI capabilities into nursing contributions through selective delegation and prompting; output verification and contextualization; and feedback, diffusion, and implementation embedding.

#### 3.4.1. Selective Delegation and Prompting

The subcategory focuses on how nurses decided what to assign to GenAI and how they communicated task requirements. Participants delegated routine, text‐based, and information‐processing work while retaining responsibility for clinical interpretation and final decisions. When initial responses were too general, they iteratively refined prompts by supplying clearer instructions and relevant contextual information.

N16: “The department often requires nurses to create PPTs, consult literature, and write book reports. I only need to give the GenAI the corresponding instructions, and it can respond quickly.”

N13: “When I first used ChatGPT, the responses looked correct, but they were quite general and difficult to directly apply. Later, I learned some prompt engineering skills. By providing more detailed instructions and patient information, the responses became more specific.”

#### 3.4.2. Output Verification and Contextualization

The subcategory relates to how nurses handled GenAI outputs during actual use. Interviewees reported treating GenAI‐generated content as provisional material that required checking, calibration, and adaptation before it could be incorporated into clinical, educational, or managerial work. This process involved comparing outputs with professional evidence, aligning them with patient circumstances and local workflows, and revising them according to nurses’ own judgment.

N14: “It doesn’t know your true intentions or understand your specific context and actual needs, so the content it generates often doesn’t quite fit the situation. I subsequently adjusted my approach, usually taking the GenAI‐generated content as a starting point and refining it based on my own judgment.”

#### 3.4.3. Feedback, Diffusion, and Implementation Embedding

The subcategory focuses on activities that extended beyond individual nurse–GenAI interaction. Participants described reporting system limitations to developers, sharing useful tools and practices with colleagues, and supporting integration into departmental workflows. These activities enabled local experience to inform system improvement and helped transform GenAI from an individual aid into a collectively supported practice resource.

N4: “Current large language models still have limitations in areas such as image recognition, and sometimes they simply do not seem intelligent enough. These issues need to be reported to development teams in a timely manner to support continuous improvement and optimization of system functions.”

N15: “Our hospital often requires nurses to participate in health education video competitions. I used a GenAI tool to generate videos, and the results were very effective. I shared this useful website with my classmates and colleagues, and they all found it helpful.”

### 3.5. Conditional Outcomes and Adaptive Co‐Evolution

This domain captures both the consequences of nurse–GenAI interaction and the feedback through which those consequences influenced subsequent collaboration. It comprised value outcomes and adaptive co‐evolution.

#### 3.5.1. Value Outcomes

##### 3.5.1.1. Value Co‐Creation

The subcategory focuses on the positive consequences generated when nurses used GenAI in ways that were aligned with nursing work and professional judgment. Participants reported that GenAI reduced repetitive and time‐consuming workload, particularly in documentation, literature searching, and teaching preparation. This reduction in routine work allowed nurses to redirect time and attention toward relational care, including patient communication, emotional support, family collaboration, and team coordination. Some participants also described GenAI as expanding nurses’ professional roles by creating opportunities to participate in digital health innovation, develop new competencies, and engage more actively in technology‐enabled nursing practice.

N17: “The nursing‐specific large language model can help summarize important information about the patients I manage during my shift and automatically generate handover notes. I just need to check the content and make sure everything is correct. It really reduces the workload of nursing documentation.”

N14: “After using GenAI, I feel that nurses are no longer only clinical care providers. We can also participate in digital health projects and contribute our nursing experience to the development of these tools.”

##### 3.5.1.2. Value Destruction

The subcategory relates to the negative consequences that emerged when GenAI outputs, work processes, or patterns of use were insufficiently controlled. Participants noted that inaccurate or inappropriate GenAI recommendations could threaten patient safety if accepted without professional verification. GenAI could also increase workload by requiring repeated checking, prompting, correction, and refinement, or by creating expectations that nurses should complete more work because efficiency had improved. In addition, several participants expressed concern that long‐term reliance on GenAI might weaken independent searching, clinical reasoning, and professional judgment, as nurses increasingly delegated thinking‐intensive tasks to the system.

N3: “I had to check everything myself and keep giving it new instructions to revise and improve the output. Sometimes I needed several rounds of conversation before getting something usable. Although GenAI was helping me, the repeated process of guiding and correcting it was actually quite exhausting.”

M7: “Although GenAI can speed up nursing documentation and PPT preparation, it actually feels like adding another whip—forcing nurses to take on even more work. With massive amounts of data and information pouring in, their workload has increased instead of decreased.”

M7: “Since I started using GenAI, I feel that I have become more dependent on it. In the past, I would carefully read research articles line by line and check the latest guidelines and professional evidence myself. But now, with various large language models and medical GenAI tools available, I often upload papers directly to GenAI instead of reading them in depth. I feel that GenAI has reduced my motivation to think independently.”

#### 3.5.2. Adaptive Feedback and Nurse–GenAI Co‐Evolution

The subcategory focuses on how experience with GenAI reshaped later patterns of use. Participants reported recalibrating trust, redefining task boundaries, and adjusting prompting and verification strategies after encountering both useful and unreliable outputs. At the organizational level, accumulated experience also informed changing expectations for system design, training, governance, and workflow integration, making value co‐creation a recursive rather than linear process.

N14: “When I first started using GenAI, I had very high expectations… However, after using it for a while, I realized that GenAI is not human; no matter how advanced it is, it has limitations. Now, I view GenAI more as a ‘strategist’—it offers ideas and points of reference, but the final decisions and adjustments remain up to me.”

## 4. Discussion

This study developed a theoretical framework showing that nurse–GenAI value co‐creation emerges through situated engagement conditions, professional orchestration in practice, and conditional outcomes and adaptive co‐evolution. The findings position nurses as active agents who determine when, how, and to what extent GenAI can contribute to nursing work, while also revealing that the same technology may generate value co‐creation or value destruction depending on task fit, clinical risk, user capability, and organizational safeguards.

### 4.1. Comparison of the N‐GAIVC Framework With Existing Theoretical and Conceptual Perspectives

The N‐GAIVC framework shares conceptual ground with four related perspectives. Connectivism views learning as the formation of connections among people, information resources, and digital technologies [26], which resonates with feedback, diffusion, and implementation embedding in the N‐GAIVC framework. The Dual Zone of Proximal Development (ZPD) framework conceptualizes GenAI as a collaborative partner whose support is calibrated to the learner’s cognitive readiness and motivational willingness [27]. Similarly, the N‐GAIVC framework involves an iterative cycle of delegation, prompting, verification, contextualization, and feedback, calibrated by nurses according to clinical tasks, risks, and professional judgment. Transactive memory systems theory emphasizes knowing where specialized knowledge resides and how it can be accessed [28], which parallels nurses’ appraisal of GenAI fit, recognition of its capability boundaries, and selective task delegation. Finally, previous research on user–GenAI value co‐creation conceptualizes value co‐creation as a dynamic process shaped by iterative human–GenAI interaction, responsible use, feedback, and multiple individual, technological, and contextual factors [29], which is consistent with the processual structure of the N‐GAIVC framework.

Compared with these theories, the N‐GAIVC framework provides a nursing‐specific, action‐oriented, and processual explanation of human–GenAI collaboration. Connectivism explains how learning and knowledge develop through connections within networks, but it does not specify how GenAI‐generated knowledge is evaluated and safely translated into high‐risk professional practice [26]. The N‐GAIVC framework addresses this limitation by showing how nurses verify, contextualize, diffuse, and embed GenAI outputs within clinical and organizational workflows. The Dual‐ZPD framework focuses on how AI can scaffold learners’ cognitive readiness and motivational engagement, whereas the N‐GAIVC framework positions nurses as the principal orchestrators who calibrate GenAI use according to task suitability, clinical risk, professional judgment, and human‐centered care [27]. Transactive memory systems theory explains how human group members coordinate distributed expertise by knowing where knowledge resides, but it does not account for collaboration with a nonhuman system whose outputs may be uncertain or contextually inappropriate [28]. The N‐GAIVC framework therefore incorporates verification, capability‐boundary appraisal, selective delegation, and professional accountability into this coordination process. Most distinctively, it conceptualizes nurse–GenAI collaboration as a conditional and recursive process linking situated engagement conditions, professional orchestration, and value outcomes. Value co‐creation or value co‐destruction subsequently reshapes task boundaries, capabilities, workflows, governance arrangements, and further nurse–GenAI adaptation. This processual explanation extends current discussions of AI‐augmented nursing practice and healthcare value co‐creation by showing how nurses’ GenAI use is shaped through ongoing interaction with clinical work over time [30–32].

The effective implementation of nurse–GenAI value co‐creation depends on institutional commitment to GenAI governance. In China, its implementation faces several interrelated challenges: (1) Under‐representation of nursing needs in GenAI development. Hospital information systems and clinical GenAI often prioritize physician workflows, leaving nursing‐specific needs inadequately addressed. As a result, nursing populations may face challenges when adopting GenAI tools, highlighting the need for nursing‐oriented development and context‐sensitive implementation strategies to support responsible GenAI integration [30, 33]. (2) Effective nurse–GenAI integration requires organizational support, governance and accountability frameworks, and active nursing involvement to ensure responsible use while preserving human‐centered nursing practice [34–36]. (3) Clinical workload constraints. China faces significant nursing workforce shortages, resulting in substantial time constraints among nurses. Because general‐purpose GenAI requires meticulous verification to ensure patient safety, this double‐checking process introduces an additional administrative burden.

Misalignment between nurses, GenAI capabilities, and organizational contexts may lead to value destruction, highlighting the need for comprehensive governance strategies to promote responsible GenAI use in nursing practice. First, clinical safety risks arise from GenAI hallucinations and contextual limitations, as GenAI may generate plausible but inaccurate outputs in healthcare settings [37]. Repeated exposure to such outputs may further increase the risk of automation bias, potentially weakening nurses’ independent critical judgment under time pressure. Second, ethical accountability represents another critical consideration. Existing studies emphasize that nurses need competencies in GenAI output verification, ethical accountability, risk awareness, and responsible decision‐making in GenAI‐assisted practice [9]. However, the distributed allocation of liability across developers, healthcare institutions, and nurses creates uncertainty regarding responsibility and post‐deployment governance, contributing to technological anxiety and reluctance toward GenAI adoption. Third, concerns regarding data privacy and security may undermine trust in nurse–GenAI collaboration. Potential data leakage and broader privacy risks represent critical barriers to trustworthy GenAI‐enabled healthcare integration, as concerns about patient privacy may undermine confidence in GenAI‐supported healthcare systems [38]. At the broader regulatory level, the European Union’s Artificial Intelligence Act (EU AI Act) classifies certain medical AI systems as high‐risk and requires conformity assessments to enhance safety, transparency, and accountability [39]. However, risk‐based definitions of GenAI “trustworthiness” may not fully capture the contextual and relational dimensions of trust that emerge during human–GenAI interactions [6].

### 4.2. Transferability and Limitations

This study has several limitations that should be considered when assessing the transferability of its findings. (1) Patient perspectives were not formally included in the analysis. Consequently, the framework primarily explains value co‐creation and value destruction from the nursing perspective and may not fully capture how patients perceive or influence nurse–GenAI collaboration. Future studies should incorporate patient voices to provide a more comprehensive understanding of the nurse–patient–GenAI triad. (2) This study was conducted in the Chinese healthcare context, which may limit the direct transferability of its findings to other healthcare systems. The proposed model is intended to provide transferable conceptual insights rather than statistically generalizable findings. Its central mechanisms—including appraising GenAI fit, safe‐use scaffolding, professional orchestration, conditional value outcomes, and adaptive co‐evolution—may provide useful concepts for examining nurse–GenAI collaboration in other settings. However, their specific manifestations may be shaped by individual characteristics and GenAI literacy, organizational leadership and culture, and alignment with established clinical workflows [40–42]. Multisite and cross‐national studies are therefore needed to examine whether these categories and relationships recur in other settings, identify the proposed model’s boundary conditions, and determine whether contextual adaptations are required. (3) Given the rapid evolution of GenAI, the findings represent nurse–GenAI collaboration during a particular stage of technological development. Longitudinal research is needed to examine how situated appraisal, professional orchestration, value co‐creation and value destruction, and recursive nurse–GenAI co‐evolution change as GenAI systems become more capable and acquire greater clinical autonomy.

### 4.3. Implications for Nursing Management and GenAI Governance

Nurse managers should support the introduction of GenAI through task‐specific, clinically governed implementation rather than broad, technology‐led deployment. Decisions about where and how GenAI is used should be made with frontline nurses and should preserve nursing control when clinical judgment, professional accountability, or the therapeutic relationship is central. The managerial role therefore extends beyond encouraging adoption to establishing conditions in which nurses can use GenAI selectively and safely.

Successful adoption also depends on workforce preparation and organizational support. The nursing workforce requires GenAI literacy and the ability to critically evaluate and appropriately integrate generated outputs into practice [33, 41]. Nurse managers and educators should support structured GenAI education that combines foundational knowledge, simulation‐based learning, supervised clinical application, and continuing professional development [9, 30]. Training alone, however, is insufficient. Sustainable collaboration requires institutional arrangements that coordinate relevant actors and support implementation in routine practice. Organizations should therefore provide accessible, approved GenAI systems and technical support that address nursing users’ digital literacy, access needs, and workflow requirements [33, 36].

GenAI governance should be embedded in everyday nursing management rather than treated as a one‐time approval exercise. Human‐in‐the‐loop protocols should clearly establish nurses’ decision‐making authority, responsibilities for verifying GenAI outputs, and procedures for escalating unsafe or uncertain recommendations. Frontline nurses should also have accessible channels for reporting problems and contributing to workflow and system improvement. Governance reviews should consider whether expected benefits are being achieved without introducing disproportionate workload, dependence, or threats to professional judgment and relational care. Evidence from clinical use should then inform continuing adjustments to training, workflows, use boundaries, and organizational safeguards.

## 5. Conclusions

This study shows that N‐GAIVC is shaped by situated engagement conditions, enacted through professional orchestration in practice, and recursively refined through conditional outcomes and adaptive co‐evolution. The framework highlights nurses as active agents who define task boundaries, verify and contextualize outputs, and embed GenAI within nursing workflows. It also shows that GenAI may generate both value co‐creation and value destruction depending on task fit, clinical risk, user capability, and organizational safeguards. These findings provide a theoretical basis for responsible GenAI integration in nursing.

## Author Contributions

Yingying Jia: writing–original draft, validation, methodology, formal analysis, data curation, and conceptualization. Wei Zhu: writing–review and editing, validation, formal analysis, and data curation. Yan Cai: writing–review and editing, validation, visualization, software, formal analysis, and data curation. Yan Jiang: validation, writing–review and editing, methodology, supervision, and project administration.

## Funding

This work was supported by the National Natural Science Foundation of China (Grant No. 72574154).

## Disclosure

The funder had no role in the study design, data collection, analysis, interpretation of data, or writing of this manuscript.

## Consent

The authors have nothing to report.

## Conflicts of Interest

The authors declare no conflicts of interest.

## Supporting Information

Additional supporting information can be found online in the Supporting Information section.

## Supporting information


**Supporting Information** Appendix A: Data collection materials and grounded theory coding procedures.

## Data Availability

Data are available upon request due to privacy/ethical restrictions.
