# Supplementary material for: Value Co‐Creation Between Nurses and Generative Artificial Intelligence: A Grounded Theory Study
Source: J Nurs Manag. 2026 Aug 28;2026:8980490. doi: 10.1155/jonm/8980490 (PMC13523069; doi:10.1155/jonm/8980490)
Supplement: Supplementary file 1 [file JONM-2026-8980490-s001.docx]

# **Supplementary Table S1. Reference topic guide.**

| **Group** | **Topic** |
| --- | --- |
| Clinical nurse | 1. Could you describe your experiences with using GenAI in your nursing practice? 2. Can you describe a specific experience where you used GenAI to support your work? What happened during this process? 3. How do you evaluate and handle the information generated by GenAI in your work? 4. In your experience, how has GenAI influenced your nursing practice? 5. What challenges have you encountered when working with GenAI, and how have you responded to them? 6. From your perspective, what changes or support are needed to better integrate GenAI into nursing practice? |
| Nurse manager | 1. Could you describe your experiences with applying GenAI in nursing management? 2. Can you describe a specific example of using GenAI in departmental management, quality improvement, or team development? 3. How do you perceive the role of GenAI in nursing management? 4. What factors influence whether GenAI can be effectively used in nursing management? 5. What challenges have you encountered when introducing or using GenAI within your department? 6. What organizational changes, leadership strategies, or resources do you think are needed to facilitate the effective integration of GenAI into nursing practice? |

# **Supplementary Table S2. Search terms for retrieving social media discussions on nurses and GenAI.**

| **Category** | **Search terms** |
| --- | --- |
| Nursing-related terms | nurse, nursing, clinical nurse, registered nurse, nurse practitioner, nursing staff, bedside nurse |
| GenAI-related terms | generative AI, GenAI, large language model, ChatGPT, GPT, GenAI assistant, GenAI chatbot |

To facilitate data triangulation, online textual data regarding nurses’ GenAI usage were collected. (1) Data platforms. We selected four widely used Chinese online platforms (Weibo, Zhihu, REDnote, and DXY.cn) to construct a comprehensive dataset. Data were collected during the period from January 1, 2022, to April 1, 2026, corresponding to the emergence and increasing adoption of GenAI in healthcare. (2) Keywords. Keywords were developed iteratively. An initial literature-derived list (e.g., “nurse GenAI”) was expanded with commonly used expressions identified from preliminary analysis and validated by two clinical nurses. Our search strategy combined the term for “Nurse” with primary keywords related to GenAI applications and collaboration. (3) Data scraping and screening. Using Python-based tools, we collected publicly accessible and anonymized online texts in accordance with platform policies. Included texts met two criteria: authored by self-identified Chinese nurses or containing clear contextual information indicating nursing-related experiences in China; and detailed personal experiences related to GenAI use, human-GenAI interaction, or technology-related experiences. Duplicates, advertisements, and spam were excluded. (4) Data preprocessing. Following automated deduplication, two researchers independently performed a manual review against the inclusion criteria, resolving discrepancies through discussion or consultation with a third researcher. During manual review, nurse identity was verified via user profiles or in-text self-identification, while experiential relevance required first-person narratives of GenAI integration.

# **Supplementary Table S3. Illustrative open-coding audit trail**

| **Source** | **Raw data excerpt** | **Initial concept coding** | **Open category** |
| --- | --- | --- | --- |
| O1 | Health informatics and nursing informatics are common in medical and nursing schools. Having a medical or nursing degree, together with a willingness to work with GenAI, is a clear advantage. | A03 Interdisciplinary medical/nursing and GenAI background as a career advantage | B01 GenAI literacy and interdisciplinary competency development |
| N6 | The GenAI guide for pediatric influenza nursing care is detailed and comprehensive. | A05 GenAI provides comprehensive nursing guidance | B02 GenAI-supported nursing knowledge |
| N10 | Qianwen explains nursing care across different stages of the disease course very clearly. | A06 GenAI explains stage-specific nursing processes | B02 GenAI-supported nursing knowledge |
| N1 | Although GenAI can assist diagnosis, medical decision-making must still integrate patients' individual differences, emotional care needs, and complex clinical conditions. For example, tactile feedback and emergency response during surgical procedures rely on nurses' experiential knowledge. | A07 GenAI-assisted diagnosis; A08 Complex decision-making relies on clinical experience; A09 GenAI lacks tactile feedback and emergency-response capacity | B03 Human-GenAI decision boundaries and professional responsibility |
| N10 | In the future, a GenAI hospital could be built, with physicians, nurses, and pharmacists replaced by machines. It could operate 24 hours a day, and programmed procedures could reduce concerns about insurance-related penalties. | A11 Imagining a fully automated GenAI hospital; A12 Machines provide around-the-clock services; A13 Programmed management reduces compliance risks | B04 Medical service automation and organizational restructuring |
| O2 | Human progress is marked by the ability to use tools well, rather than being enslaved by them. If this order is reversed, a GenAI crisis may not be far away. | A16 Humans should retain agency in technology use; A17 Guarding against GenAI domination | B05 Positioning GenAI as a tool and preserving human agency |
| N4 | Excellent people become better with GenAI; foolish people become more foolish with GenAI; and bad actors become worse with GenAI. | A18 GenAI amplifies users' existing capabilities; A19 GenAI amplifies users' moral orientations | B05 Positioning GenAI as a tool and preserving human agency |
| N8 | GenAI is essentially a tool. It is not GenAI that eliminates people; rather, people who cannot use GenAI may be eliminated. | A20 Positioning GenAI as a tool; A21 GenAI-use capability affects occupational competitiveness | B05 Positioning GenAI as a tool and preserving human agency |
| N10 | Using GenAI tools appropriately while maintaining independent thinking is the core competency. | A22 Appropriate use of GenAI; A23 Maintaining independent thinking | B05 Positioning GenAI as a tool and preserving human agency |
| N12 | In eldercare, the core work is not fixed routines but accurately detecting subtle emotional fluctuations and physiological changes, and soothing patients through humanistic care. Interpersonal trust and delicate emotional companionship cannot be reproduced through preset code. | A33 Care work is difficult to replace; A34 Identifying subtle emotional and physiological changes; A35 Soothing patients through humanistic care; A36 Interpersonal trust cannot be encoded or replicated | B06 Humanistic care and relational value |
| N11 | What is the essential difference between intelligence and wisdom? When algorithms enter emotional companionship, moral judgment, and even cognitive collaboration, what remains uniquely human? | A37 Reflecting on boundaries in human-GenAI interaction; A38 Distinguishing machine intelligence from human wisdom; A39 Concern about algorithmic intrusion into core human domains | B07 Human-GenAI boundaries and trust ethics |
| O2 | With the help of a colleague in a nursing school, Yang developed a GenAI-based solution to prevent falls among older adults. For two years, she tried to develop the product while also completing research and teaching responsibilities, but this was unsustainable. She eventually took leave and, 18 months later, resigned to found OK2StandUp, which serves nursing homes and other healthcare clients. | A78 GenAI supports fall-risk prevention; A79 Nursing research is translated into a GenAI product; A80 Nurses engage in GenAI entrepreneurship | B08 Nurse-led GenAI innovation translation |
| N6 | Expanding the boundaries of GenAI also brings new challenges. When GenAI enters healthcare, justice, and other ethically sensitive domains, how can technical efficiency be balanced with humanistic care? When personalized services rely on large volumes of private data, how can precision adaptation be reconciled with information protection? | A117 Balancing technical efficiency and humanistic care; A118 Balancing personalization and privacy protection; A119 Restricting GenAI entry into ethically sensitive domains | B07 Human-GenAI boundaries and trust ethics |
| M7 | The expansion of GenAI boundaries is essentially a process in which humans redefine the boundaries of capability and trust. Ultimately, application scenarios that enhance efficiency while preserving human warmth will become new markers of harmonious coexistence between technology and humanity. | A120 Human intention determines GenAI application boundaries; A121 Redefining capability and trust boundaries; A122 Balancing efficiency with humanistic warmth | B07 Human-GenAI boundaries and trust ethics |
| M8 | Technological bottlenecks and ethical dilemmas remain important, including immature tactile-feedback technology, black-box medical decision-making, and legal gaps in liability for accidents. These issues shape the pace of the GenAI revolution. | A126 Immature tactile-feedback technology; A127 Black-box problems in medical decision-making; A128 Unclear liability for GenAI-related accidents | B09 GenAI governance norms and accountability implementation |
| O3 | MedLM can address pain points in healthcare, such as heavy documentation workloads and inefficient information retrieval, allowing healthcare workers to devote more energy to patient care. | A134 GenAI reduces documentation workload; A135 GenAI improves information-retrieval efficiency; A136 Time is redirected to patient care | B10 Work efficiency improvement and workload reduction |
| O4 | As GenAI plays an increasingly important role in clinical practice, concerns are growing that outsourcing clinical tasks and reasoning to GenAI may cause skill loss or degradation, inheritance of GenAI errors or biases, or failure to develop necessary competence. | A142 Outsourcing cognitive tasks; A143 GenAI leads to skill degradation; A144 Inheriting GenAI errors and biases; A145 Impaired development of core competencies | B11 GenAI dependence and professional skill degradation |
| M8 | Beyond procedural work and image interpretation, the critical thinking required for clinical reasoning in diagnosis, decision-making, and patient management may also be weakened through interaction with GenAI. | A146 GenAI reduces clinical vigilance; A147 GenAI weakens visual analytic ability; A148 GenAI weakens clinical reasoning; A149 GenAI weakens critical thinking | B11 GenAI dependence and professional skill degradation |
| O11 | When GenAI becomes embedded in clinical practice, new risks emerge: vigilance may decline, skills may deteriorate, and deeper reliance on GenAI may reduce nurses' confidence in unaided judgment. These concerns are especially salient in nursing education. Trainees may rely on GenAI before mastering core competencies; if the technology fails, they may lack a dependable foundation and miss experiences needed to develop clinical intuition and judgment. | A150 GenAI shares high-burden clinical work; A151 Overreliance reduces confidence in independent judgment; A152 Premature GenAI use leads to skill deficits | B11 GenAI dependence and professional skill degradation |
| O13 | Protecting nurses' skills and critical thinking in GenAI-enabled settings has no single solution. Skill degradation, overreliance, and loss of vigilance may vary by algorithm, specialty, and context. Safeguards may include brief GenAI-off or GenAI-delayed steps in clinical workflows to recalibrate vigilance and assess unaided performance. In training or skill maintenance, GenAI interpretations could be displayed only after nurses enter their own findings. Clearer boundaries are also needed: GenAI should handle rule-based, high-volume, low-ambiguity tasks, allowing nurses to focus on contextual, ambiguous, and high-risk decisions. Such boundaries can reduce automation bias and automation neglect. | A153 Technology failure exposes competence gaps; A154 Setting GenAI-off or GenAI-delayed steps; A155 Making independent judgments before viewing GenAI output; A156 Allocating human-GenAI responsibilities by task risk; A157 Preventing automation bias and automation neglect | B11 GenAI dependence and professional skill degradation |
| M1 | Whatever strategy is adopted, its foundation must be nurses' sustained capacity to evaluate the trustworthiness of any GenAI tool. Choices made now about GenAI design, integration, and training will determine whether these systems enhance professional practice or quietly erode the core skills that define it. | A158 Continuously evaluating GenAI trustworthiness; A159 Recognizing the clinical detection value of GenAI; A160 Guarding against GenAI-induced changes in healthcare behavior; A161 Protecting professional competence through workflow design; A162 GenAI training determines capability enhancement or erosion | B11 GenAI dependence and professional skill degradation |
| O5 | Current trustworthy GenAI frameworks rely on explainable GenAI, which explains to clinical nurses why a model makes a recommendation. In multi-agent environments, this paradigm has a critical limitation: explanations are inherently descriptive and retrospective. | A168 Trustworthy GenAI relies on explainability mechanisms; A169 Multi-agent explanations are retrospective; A170 Post hoc explanations cannot support real-time intervention | B12 GenAI explainability and black-box risks |
| O6 | Contestability, rather than an optional extension of explainability, is a necessary design requirement for trustworthy multi-agent algorithmic nursing systems. Borrowing from legal theory, contestability refers to clinicians' ability to challenge GenAI outputs, requiring system-level rights to demand justification, intervene in decision processes, and initiate review, correction, or override mechanisms. Unlike explainability, which emphasizes one-way information flow and asks why GenAI reached a conclusion, contestability emphasizes a two-way interaction loop that asks how humans can challenge GenAI and change its decisions. | A171 Treating contestability as a necessary requirement for trustworthy GenAI; A172 Granting clinicians the right to question, correct, and override GenAI; A173 Establishing a bidirectional human-GenAI interaction loop | B13 GenAI contestability and clinical intervention authority |
| O7 | In multi-agent healthcare, contestability means that humans can identify whether an individual agent's recommendation or the aggregation logic among agents is wrong and can use tools to correct it. Human nursing planners can accept, reject, revise, or add arguments; these modifications are reinjected into the system and confidence is recalculated, ensuring that human intervention materially changes the final output. Explainability without contestability deprives clinical nurses of agency and turns them into passive executors. | A174 Identifying erroneous agents and aggregation logic; A175 Reviewing GenAI through structured disagreement; A176 Human intervention materially changes system outputs; A177 Restoring clinicians' agency | B13 GenAI contestability and clinical intervention authority |
| N15 | The spread of GenAI may still be constrained by institutional inertia, existing norms, unclear legal liability, and misaligned incentives. Regulatory systems must evolve to accommodate higher levels of autonomy, including prescribing authority for autonomous systems, documentation standards for autonomous clinical decisions, and appropriate payment and reimbursement mechanisms. | A180 GenAI improves healthcare accessibility; A181 Organizational inertia and legal liability hinder implementation; A182 Regulatory, prescribing, and payment systems require adaptation | B14 Institutional adaptation and scaled implementation of medical GenAI |
| O8 | The efficiency revolution allows GenAI to replace repetitive work and return healthcare professionals to clinical value. Quality improvement is promoted through algorithmic assistance that reduces missed or incorrect diagnoses and supports standardization. Care models are reconstructed from nurse-led practice toward human-machine collaboration, enabling high-quality medical resources to reach primary care settings. | A183 GenAI replaces repetitive work; A184 GenAI promotes standardized diagnostic and treatment quality; A185 Healthcare workers return to high-value clinical tasks; A186 Forming a clinical, data, and research cycle | B15 Clinical implementation of GenAI and nursing service expansion |
| M2 | Current methods for evaluating chatbots do not capture the complexity of interaction with human users. | A201 Existing evaluation methods overlook the complexity of real human-GenAI interaction | B16 GenAI evaluation and real-world interaction validation |
| N17 | By analyzing large volumes of data, GenAI helps nurses make more informed decisions. GenAI-driven automated patient-monitoring systems can track and analyze patients' vital signs and conditions in real time, reducing nurses' continuous monitoring burden. | A202 GenAI supports nursing decision-making; A203 Automated monitoring reduces nurses' workload; A204 GenAI provides personalized nursing education; A205 GenAI supports smart ward services | B17 GenAI-enabled whole-process nursing |
| M2 | When health data are used, GenAI systems may access sensitive personal information; therefore, strong legal and regulatory frameworks are needed to protect privacy, security, and integrity. Data transparency, documentation, risk management, and external validation are essential. GenAI models may fail to represent population diversity accurately, leading to bias, inaccuracy, or failure. | A206 GenAI uses sensitive health information; A207 Transparent documentation, risk management, and external validation are needed; A208 Insufficient data representativeness leads to algorithmic bias | B09 GenAI governance norms and accountability implementation |
| O9 | You do not know why it acts as it does: it is a black box. It may go off track because, when focused only on its target, an agent may do something completely unexpected. This creates a responsibility vacuum: everyone thinks someone else is at fault, and in the end no one is accountable. The more capable the agent is, the greater the potential harm if it is misused. | A218 Agentic decision-making processes are opaque; A219 Goal misalignment leads to unexpected behavior; A220 Agents create a responsibility vacuum; A221 Agents may be maliciously exploited | B09 GenAI governance norms and accountability implementation |
| M3 | Nursing practitioners need a solid foundation in medical knowledge. They also need broad technical skills, including computer operation, data analysis, and GenAI principles and applications. Nurses must continuously improve their competencies and adapt to technological change. | A231 Integrating medical, nursing, and GenAI knowledge; A232 Using GenAI for personalized rehabilitation; A233 Conducting interprofessional teamwork; A234 Continuously learning and adapting to technological change | B01 GenAI literacy and interdisciplinary competency development |
| M5 | GenAI will mainly augment rather than replace nurses. Future nurses will be increasingly freed from efficiency-oriented repetitive work and redirected toward inefficient but high-value domains requiring breakthrough thinking. Human warmth itself may become a high-end service. Work requiring deep empathy, complex interpersonal mediation, and warm companionship, such as advanced nursing, psychotherapy, and personalized education, will become increasingly important. | A245 GenAI augments rather than replaces humans; A246 Humans shift toward breakthrough innovation; A247 GenAI may develop companionship relationships; A248 Human warmth and deep empathy become scarce values | B06 Humanistic care and relational value |

**Note.** O1, O2, etc. denote online/social-media textual data; N1, N2, etc. denote clinical nurses; and M1, M2, etc. denote nursing managers.

# **Supplementary Table S4. Axial coding of the 14 subcategories into six main categories**

| **Main category** | **Subcategories** | **Paradigmatic role** | **Analytic relationship** |
| --- | --- | --- | --- |
| MC1. Engagement drivers | SC1. Workload and efficiency needs  SC2. Perceived GenAI affordances and role opportunities | Causal conditions | Explains why nurses began to consider or engage with GenAI. |
| MC2. Appraising GenAI fit | SC3. Task codifiability and clinical risk  SC4. Agency, accountability, and explainability  SC5. Human-centered care fit | Contextual conditions | Determines whether, when, and to what extent GenAI is considered appropriate for a particular nursing task. |
| MC3. Safe-use scaffolding | SC6. GenAI literacy and critical evaluation  SC7. Training, infrastructure, and governance  SC8. Nursing-specific data and real-world validation | Intervening conditions | Enables or constrains nurses’ ability to use GenAI safely and responsibly. |
| MC4. Professional orchestration | SC9. Selective delegation and prompting  SC10. Output verification and contextualization  SC11. Feedback, diffusion, and implementation embedding | Action/interaction strategies | Describes how nurses transform general GenAI outputs into clinically and contextually appropriate nursing contributions. |
| MC5. Value outcomes | SC12. Value co-creation  SC13. Value destruction | Consequences | Shows that GenAI may generate value co-creation or value destruction depending on task fit, safeguards, and professional orchestration. |
| MC6. Adaptive co-evolution | SC14. Adaptive feedback and nurse–GenAI co-evolution | Feedback process | Explains how previous outcomes reshape subsequent GenAI engagement and organizational implementation. |

# **Supplementary Table S5. Selective coding and integration around the core category**

| **Core category** | **Integrative domain** | **Main category** | **Relationship with the core category** |
| --- | --- | --- | --- |
| Nurse-led GenAI orchestration for situated nursing value co-creation | I. Situated engagement conditions | MC1. Engagement drivers | Identifies the practice pressures and perceived affordances that initiate nurses’ engagement with GenAI. |
|  |  | MC2. Appraising GenAI fit | Defines the task-related, clinical, ethical, and relational boundaries within which GenAI can be used. |
|  |  | MC3. Safe-use scaffolding | Provides the individual and organizational capabilities, safeguards, and validation conditions required for responsible collaboration. |
|  | II. Professional orchestration in practice | MC4. Professional orchestration | Represents the central action/interaction mechanism through which nurses translate GenAI outputs into contextually appropriate nursing contributions. |
|  | III. Conditional outcomes and adaptive co-evolution | MC5. Value outcomes | Captures the value co-creation and value destruction that may result from aligned or misaligned orchestration. |
|  |  | MC6. Adaptive co-evolution | Explains how prior outcomes reshape subsequent engagement, boundaries, capabilities, governance, and implementation. |
